# Knowledge and practices of youth awareness on death and dying in school settings: a systematic scoping review protocol

**DOI:** 10.1186/s13643-024-02635-9

**Published:** 2024-08-24

**Authors:** Emilie Allard, Clémence Coupat, Sabrina Lessard, Noémie Therrien, Claire Godard-Sebillotte, Dimitri Létourneau, Olivia Nguyen, Andréanne Côté, Gabrielle Fortin, Serge Daneault, Maryse Soulières, Josiane Le Gall, Sylvie Fortin

**Affiliations:** 1 Faculty of Nursing, Université de Montréal, Montréal, QC Canada; 2 Research Center, Centre Intégré Universitaire de Santé et de Services Sociaux du Nord-de-l’Île-de-Montréal (CIUSSS NIM), Montréal, QC Canada; 3 Centre for Research and Expertise in Social Gerontology, Centre Intégré Universitaire de Santé et de Services Sociaux Centre-Ouest-de-l’Île-de-Montréal (CIUSSS-CCOMTL), Montréal, QC Canada; 4 Department of Anthropology, Université de Montréal, Montréal, QC Canada; 5 Faculty of Medicine, Université de Montréal, Montréal, QC Canada; 6 Division of Geriatric Medicine, McGill University Health Centre (MUHC), Montréal, QC Canada; 7 Division of Geriatrics, Department of Medicine, McGill University, Montréal, QC Canada; 8 MUHC Research Institute, Montréal, QC Canada; 9 Department of Family and Emergency Medicine, Université de Montréal, Montréal, QC, Canada; 10 Palliative Care Services, CIUSSS NIM, Montréal, QC Canada; 11 School of Social Work and Criminology, Université Laval, Québec, QC Canada; 12 Research Center, CHU de Québec-Université Laval, Québec, QC Canada; 13 Research Center, Montreal University Institute of Geriatrics, Montréal, QC Canada; 14 School of Social Work, Université de Montréal, Montréal, QC Canada; 15 Research Center SHERPA, CIUSSS-CCOMTL, University Institute on Immigration, Diversity, and Health, Montréal, QC Canada; 16 Montreal University Centre for Ethnic Studies (CEETUM), Montréal, QC Canada; 17 Department of Pediatrics, Faculty of Medicine, Université de Montréal, Montréal, QC Canada

**Keywords:** Death literacy, Death, Dying, School, Education, Youth, Adolescent, Intervention, Systematic review

## Abstract

**Background:**

Awareness-raising and education have been identified as strategies to counter the taboo surrounding death and dying. As the favoured venue for youth education, schools have an essential role to play in informing future decision-makers. However, school workers are not comfortable addressing the subjects of death and dying, which, unlike other social issues, have no guidelines to influence awareness of these subjects in youth.

**Objectives:**

To systematically explore the knowledge and practices on raising awareness about death and dying in schools, the viewpoints of the people involved (young people, school workers; parents), and the factors that either promote or hinder awareness practices.

**Method:**

The scoping review method of Levac and Colquhoun (Implement Sci 5(1):69, 2010) will be used. Using a combination of keywords and descriptors, a body of literature will be identified through 15 databases and through grey literature searches, manual searches, consultation of key collaborators, and the list of relevant literature. Publications since 2009 will be selected if they relate directly to awareness-raising about death and dying in schools. Writings will be selected and extracted by two independent people, and conflicts resolved by consensus. The extracted data will be synthesized using a thematic analysis method. Experts from a variety of disciplines (health sciences, humanities, social sciences, and education) will be consulted to enhance the interpretation of the preliminary results. Results will be presented in narrative form and will include tables and diagrams.

**Conclusion:**

The results of this scoping review will contribute to the development of educational practices adapted to young people and to the identification of future avenues of research on awareness of death and dying.

**Supplementary Information:**

The online version contains supplementary material available at 10.1186/s13643-024-02635-9.

## Background

The recent report of the *Lancet Commission on the Value of Death* [1] reveals the uneasy relationship between the twenty-first century society, particularly in affluent countries, with death and dying, i.e. the process surrounding the death of a person, including the idea of our own death. The authors of the report emphasize that there is still much to be done to reverse people’s often negative representations of death and dying and the lack of knowledge, discomfort, anxieties, and sometimes even taboos regarding these issues. Thus, although death and dying are common and inescapable realities for all human beings, addressing these phenomena openly in Western society can be difficult, particularly since the subject is often emotionally laden and sometimes considered taboo [1, 2]. This difficulty is even more acute when dealing with children and adolescents,^1^ where factors such as age, developmental stage, personality, or religious beliefs [3–5] can shape their understanding of dying and death. What’s more, adults are afraid to broach these subjects with young people for fear of causing them suffering and anxiety as well as the fact that they may have their own anxiety about the subject [3, 6, 7].

Yet researchers have shown that young people construct their own understanding of these phenomena, within the societal and cultural context in which they grow up [8]. Young people come into contact with death and dying in various ways. They may experience bereavement directly, through the death of a close relative (grandparent, parent, friend) or companion animal. Death is also represented in the world of television, media, cartoons [9–11], books [3, 5] and video games [12].

One way to counter the taboo surrounding death and dying is through awareness-raising and education [1, 13]. Death literacy is considered to stem from experiences and learnings about death and dying that help improve individuals’ and communities’ ability to act in these situations [14]. To become death literate, it is important to support educational initiatives on the subject of death, so young people—considered as social actors and citizens of tomorrow—can be better equipped to face death, understand the situations and care involved with it, and participate in accompanying and supporting those going through these situations.

As the favoured venue for educating youth, schools can play a key role in death literacy. During a talk on end-of-life issues given by the principal investigator (PI) to elementary school children, it was observed that they appreciated being able to openly discuss their views on death and dying, which are largely influenced by their personal experiences (e.g. death of a grandparent) and social interactions (e.g. social media, friends). On the other hand, school workers say they are ill-equipped to tackle this issue with youth, not knowing what to say nor how to approach it [15]. In a socially and culturally diverse environment that includes young people of different origins, beliefs, migratory statuses, and life experiences, talking about death can be even more sensitive, since it not only involves the abovementioned taboo but also a plurality of cultural and religious beliefs surrounding these final moments of life [16]. School workers also report being concerned about how parents will react to this topic, which is considered a social taboo and is influenced by the cultural aspects, beliefs, and values held by each family.

To our knowledge, there are no resources for school workers to initiate a dialogue with students about death and dying. However, other social issues (e.g. sexual and gender identity) have been incorporated into the educational curricula in some countries, drawing on government and international guidelines [17]. While ad hoc initiatives concerning death and dying are being produced [18], the state of knowledge and practices on raising awareness about these subjects among school-aged young people needs to be clarified. This would make it possible to identify and implement actions that could support the training of school workers in addressing death and dying with youth as well as practices contributing to the death literacy of our future decision-makers.

## Goals of the review

To guide the development of cross-sectoral (education, health, and social sciences) death literacy interventions for children and staff in school settings, this systematic scoping review will explore the state of knowledge and practices in raising awareness of death and dying among young people in schools, the viewpoints of the people involved (young people, school workers, parents), and the factors that promote or hinder such awareness-raising. In fact, this type of review will make it possible to conduct an extensive, exhaustive, and comprehensive examination and analysis, including publications of a variety of methods and grey literature. This thereby enables the identification of practices that can inform the development of awareness-raising interventions.

## Methods

Levac [19] scoping review method will be used. This method comprises six steps: (1) identify the review questions, (2) identify the literature, (3) select the literature, (4) extract data, (5) report the result, and (6) consult stakeholders.

This protocol is registered with Open Science Framework (OSF) [20] and based on the Preferred Reporting Items for Systematic Reviews and Meta-Analyses extension for Scoping Reviews (PRISMA- ScR) (see supplementary file 1). As the scoping review is carried out iteratively, this protocol will serve as the starting point for documenting adjustments and changes to the method.

### Step 1: Identify the review questions

The following questions will guide the scoping review:

1. How do we raise awareness on death and dying in the school settings?
2. What are the views of young people, parents, and school workers on raising awareness about death and dying in the school settings?
3. What factors help or hinder this awareness-raising in the school settings?

### Step 2: Identify the literature

#### Inclusion and exclusion criteria

Literature that meets the population-concept-context (PCC) criteria will be included [21].

##### Population

Two types of population have been identified to answer the questions posed by the scoping review. The main population is youth in schools, i.e. children or teenagers attending elementary or high schools, who are the targets of awareness-raising practices.

Within the selected literature, the scoping review process will also focus on extracting the viewpoints of the people involved with these young people, notably parents, teachers, and other practitioners in school environments (nurse, principal, etc.). These make up our secondary population.

##### Concept

The central concept of this scoping review refers to raising awareness of death and dying, i.e. arousing interest and offering relevant, scientifically informed information to support individual and social reflection on the subject. This concept thus intersects with death education and literacy. As previously mentioned, death literacy results from individuals’ experiences and learnings, enabling them to project themselves into the future (prospection), to better understand and improve experiences around death and dying [14].

Considering the plurality of terms used to define awareness-raising, education, and literacy on death and dying, the literature include in this scoping review must report on how young people are exposed to and led to reflect on these concepts in a school setting. Dying refers to the physical, psychosocial, cultural, and spiritual processes that lead to a person’s death [22]. This concept thus incorporates care and practices, as well as the losses and bereavement associated with this period of human existence. Therefore, are included the publications on the full range of end-of-life care, including palliative care, end-of-life care, medical aid in dying, and assisted suicide. Death, the cessation of vital functions, marks the end of life and thus also the end of the dying process. As death and dying are universal social phenomena, no restrictions are placed on health status, context (natural disaster, war, other tragedies, etc.), or the age of the deceased. However, the following types of publications are excluded: those on suicide prevention, those on serious health conditions in which death or the end of life is not a central issue (e.g. chronic illness), and those discussing bereavement not related to death (e.g. divorce).

##### Context

Publications will be considered if they deal with raising awareness about death and dying explicitly and exclusively in a school setting. Given the differences in educational structures between countries, the school settings included will be all elementary and secondary education environments (or their equivalents). Excluded will be publications about informal education settings (e.g. family, daycare), postsecondary education settings, and activities taking place outside the institutional framework of a school (e.g. extracurricular or community activities).

##### Type of records

The search strategy will be limited to publication in English or French, but without restriction on the place of study. Over the last few decades, the evolution of technology has led to changes in teaching methods in Western societies. The number of writings on technology in education has boomed since 2009, reflecting the implementation and adaptation of the school environment to the digital age, the development of information technologies, the introduction of the Internet in various communities, the development of distance learning, and generational changes [23–25]. To ensure that this search reflects the challenges of contemporary social, pedagogical, and societal change, only publications from January 1, 2009, onwards will be included.

All types of literature will be considered, including primary studies of various designs (e.g. experimental, quasi-experimental, observational, qualitative, mixed), literature reviews (e.g. meta-analyses, systematic reviews, narrative reviews), grey literature (e.g. theses, research reports, models of educational practice), and theoretical publication dealing specifically with the subject of raising young people’s awareness of dying and death in the school environment. The following are excluded: blogs, media entries, personal opinions, book reviews, letters to the editor, editorials, conference abstracts, and research protocols.

### Step 3: Select the literature

#### Information sources

Four categories of information sources will be used to identify the literature.

1. *Databases*: The following databases will be surveyed: CINAHL Complete (Cumulative Index to Nursing and Allied Health Literature) (EBSCO), MEDLINE (Medical Literature Analysis and Retrieval System Online) (Ovid), EBM (Evidence-Based Medicine) Reviews Cochrane (Ovid), JBI EBP (Evidence-Based Practice) Database (Ovid), PsycINFO (Ovid), Web of Science (Clarivate), Global Health (OVID), Sociological Abstracts (ProQuest), Social Sciences Abstracts (EBSCO), Family Studies Abstracts (EBSCO), Social Services Abstracts (ProQuest), Social Work Abstracts (EBSCO), Erudit, CAIRN, and PubPsy.
2. *Grey literature*: A grey literature search will be conducted systematically in the following databases: Dissertations & Theses Global (ProQuest) and Google Scholar.
3. *Reference searching*: The reference list of the publications included in the review will be examined to find other relevant sources. The same will be done with the tables of contents of journals that have published key publications.
4. *Key authors and collaborators*: The key authors and collaborators to this project will be contacted by email to identify unindexed literature or unpublished practice guidelines, to verify the completeness of the search strategy.

#### Search strategy

In collaboration with a health sciences librarian, a literature search strategy was developed using a combination of the three concepts (see Table 1): (1) death and dying, (2) youth, and (3) school. Initially developed for the CINAHL-Complete (EBSCO) database, the search strategy was subsequently adapted for the other databases. The optimization of the search strategy by descriptors and keywords took place over a 4-month period, between January and May 2023. Keywords are searched for in titles, abstracts, and keywords, to identify publications not indexed in database thesauri. The Medical Subject Headings (MeSH) terms used for MEDLINE are presented in Table 1, and supplementary file 2 presents all the search strategies used.

**Table 1:** Concepts and keywords used on databases: MeSH used on MEDLINE

Concepts	Awareness/education about dying and death	Young people	School
**Keywords**	Death Dying Bereavement Bereft Grief Grieving Mourning Palliative care End of life Terminal care Terminally ill Hospice care Hospices Supportive care Euthanasia Medical aid in dying Assisted suicide Funerals Attitude to death Death ritual	Youth Child (*children*) Childhood (boyhood, girlhood) Boy Girl Kid Adolescence Adolescent Teenager Teen	Primary school Elementary school Secondary school High school Kindergarten Curriculum Teacher Pupils Primary education Secondary education
**MeSH terms in MEDLINE**	exp Death/ Palliative care/or Terminal care/ bereavement/or grief/ exp Hospice Care/ exp Hospices/ exp Euthanasia/ Suicide, Assisted/ Attitude to Death/or Funeral Rites	Child/ Adolescent	Schools/ Students/ School Teachers/ Teaching/ exp Curriculum

Here is the final strategy for MEDLINE database: (((exp Death/ or Palliative care/ or Terminal care/ or bereavement/ or grief/ or exp Hospice Care/ or exp Hospices/ or exp Euthanasia/ or Suicide, Assisted/ or Attitude to Death/ or Funeral Rites/) or ((Death* or Dying or Palliati* or Hospice* or Euthanasia or Bereav* or Bereft or Grief or Grieving or Mourning or Funeral* or ((Terminal* adj1 (care OR ill*)) or (suicide adj2 assist*)) or "End of life" or "Supportive care").ab,kf,ti.)) AND ((Child/ or Adolescent/) or ((Youth* or Child* or Boy* or Girl* or Kid or Kids or Adolescen* or Teen*).ab,kf,ti.)) AND ((Schools/ or Students/ or School Teachers/ or Teaching/ or exp Curriculum/) or (School* or Kindergarten* or Curriculum* or Teacher* or Pupil* or ((Education or Student*) adj1 (Primary or Secondary or Elementary)).ab,kf,ti.)) AND (limit to yr = "2009—2023")).

#### Study records

##### Data management

The literature obtained through this search strategy will be imported into the Covidence systematic review assistance tool (Veritas Health Innovation Ltd., Melbourne, Australia), which removes duplicates and allows the literature selection process to be done independently by team members.

##### Selection process

To calibrate the selection process and define the exclusion criteria, a committee, made up of several members of the research team, will select 15% of the literature randomly chosen. Selection tools will be produced following this calibration process, and the rest of the selection will be carried out by four members. The selection process will begin with a reading of each title and abstract. To be included in this first stage, a publication must be independently accepted by two people. Conflicts will be discussed and resolved by consensus, if necessary, involving a team member from outside the selection process.

The second stage of the selection process is the full-text review by two independent team members. Using five full texts, chosen for their differences (e.g. type of records, designs), a calibration process will be undergone by several team members to clarify inclusion and exclusion reasons. At this stage, reasons for exclusion will be documented. Publications deemed uncertain, and conflicts will again be discussed by the selection team, to reach a consensus resolution. A unique identifier will be assigned to the publications included at the end of the selection process.

### Step 4: Extract data

As for the selection process, the extraction will be carried out by a subgroup of the research team after a calibration process to fine-tune the extraction tool. The calibration process will be the extraction of two publications by the team members involved in the extraction process to establish agreement. After the calibration process, each publication will be extracted by one person, and the extraction will be validated by another team member. Uncertainties will be discussed as a team. Using a template built in Covidence, the following data will be extracted, if mentioned, and depending on the nature of the selected publication.

1. General data: Title, publication year, authors’ names, discipline of first author, country, type of writing (e.g. literature review, primary study, practice summary), purpose, and objectives
2. Theoretical data: The philosophical stance and frame of reference guiding the project or the practice
3. Data on interventions/practices: Type of awareness-raising practice (e.g. conference presentation, curriculum), characteristics (e.g. time, subjects), barriers and facilitators, people involved, and their characteristics
4. Methodological data: Research design, setting, sample (number, inclusion, and exclusion criteria), participant characteristics (e.g. age, grade), data collection and analysis methods, strengths, and limitations identified by the authors
5. Results data: Various stakeholders’ viewpoints on awareness-raising practices, influencing factors, consequences or impacts of the practice, and suggestions for improvement

Assessing the methodological quality of the selected literature is not a required step according to Levac [19]. In this scoping review, methodological quality will not be assessed, due to the expected diversity of publications from both research and practice models. Nevertheless, the data extracted, methods used, and transferability of the practices reported will be considered critically. During the consultation phase, partners and collaborators will be invited to comment on the results.

### Step 5: Report the results

The selection process will be illustrated using a diagram from the Preferred Reporting Items for Systematic Reviews and Meta-Analyses extension for Scoping Reviews (PRISMA-ScR) [26]. The extracted data will be analysed using the content analysis method of Miles and Huberman [27], which comprises three steps: (1) condensing the data (coding), (2) finding similarities and differences, and (3) drawing conclusions (identifying themes and subthemes). The results will be presented in narrative form, integrating results from a variety of publications, with tables and graphs to identify the specific features of each. The presentation of the results will answer the three research questions.

### Step 6: Consult stakeholders

The sixth step is deemed optional by the method designers, but given the nature of our scoping review, a great deal of time will be spent consulting external parties to identify awareness-raising practices. First, project partners and collaborators will be consulted to identify additional or unpublished texts on raising youth awareness of death and dying. The list of identified references, together with the inclusion and exclusion criteria, will be shared with them so they can suggest additional references, particularly from the grey literature. When a first version of the result synthesis is produced, it will be shared with them to obtain their view, given their experience with and expertise on the subject. Specific questions will be sent to them in writing (email) or via a telephone discussion with a member of the research team. These consultations will enhance the interpretation of the results.

## Discussion

To the best of our knowledge, no publication exists to guide the development of awareness-raising practices on death and dying in schools. This scoping review hopes to identify promising practices along with the factors influencing youth awareness-raising and the challenges associated with such practices. This project is also in line with the recommendations of the recent report of the *Lancet Commission on the Value of Death* [1], which stresses the importance of educating the population in order to transform the social view of death and dying and to recognize these phenomenon as integral parts of the human experience. The results can then be used to guide school staff in setting up educational activities in line with children’s age and development stage. The project’s conclusions will offer concrete recommendations to decision-makers in educational environments and governments on how to incorporate these themes into the educational pathways of tomorrow’s citizens.

The limitations of this scoping review include the lack of assessment of the quality of the selected literature, which may influence the recommendations that emerge. Nevertheless, the aim of this scoping review is to consider the state of knowledge and practices in the field of awareness-raising of death and dying in school settings, which does not require an assessment of the quality of the literature reviewed. The combination of multiple sources of information and types of writings is a challenge for such a systematic review but is also a source of richness. In addition to using a systematic method and complying with the PRISMA-ScR recommendations, the strengths of this scoping review lie in the quality and diversity of the research team, which includes several researchers with cross-sectoral expertise (education, health, humanities, and social sciences) complementary to the study, as well as experience in carrying out systematic knowledge synthesis. The team works closely with a librarian and with local and international collaborators and partners carrying out awareness-raising activities among the target population.

### Supplementary Information

Supplementary Material 1. Preferred Reporting Items for Systematic reviews and Meta-Analyses extension for Scoping Reviews (PRISMA-ScR) Checklist

Supplementary Material 2: Database.

## Data Availability

See OSF registration: 10.17605/OSF.IO/EHY8T.

## Footnotes

1 Hereinafter referred to as “young people” or “youth”, with the aim of being inclusive, without any judgements about age

## References

[1] Sallnow L, Smith R, Ahmedzai SH, Bhadelia A, Chamberlain C, Cong Y, et al. Report of the Lancet Commission on the value of death: bringing death back into life. Lancet. 2022;399(10327):837–84. 35114146 10.1016/S0140-6736(21)02314-X PMC8803389

[2] Fortin S, Le Gall J. Présentation: fin de vie et mourir contemporains. Anthropol Soc. 2021;45(1–2):15–24.

[3] Longbottom S, Slaughter V. Sources of children’s knowledge about death and dying. Philos Trans R Soc B Biol Sci. 2018;373(1754):20170267. 10.1098/rstb.2017.0267 PMC6053990 30012734

[4] McClement S, Stenekes S. Comment parler d’une maladie grave à un enfant ou à un adolescent. Portail Palliatif Canadien; 2023. Available from: https://www.virtualhospice.ca/fr_CA/Main+Site+Navigation/Home/Topics/Topics/Communication/Talking+with+Children+and+Youth.aspx.

[5] Mahon MM. Death in the lives of children. In: Talwar V, Harris PL, Schleifer M, editors. Children’s understanding of death: from biological to religious conceptions. New York: Cambridge University Press; 2011. p. 61–97.

[6] Hanna JR, McCaughan E, Semple CJ. Challenges and support needs of parents and children when a parent is at end of life: a systematic review. Palliat Med. 2019;33(8):1017–44. 31244381 10.1177/0269216319857622

[7] Rapa E, Hanna JR, Mayland CR, Mason S, Moltrecht B, Dalton LJ. Experiences of preparing children for a death of an important adult during the COVID-19 pandemic: a mixed methods study. BMJ Open. 2021;11(8):e053099. 34400462 10.1136/bmjopen-2021-053099 PMC8370837

[8] Ahmadi F, Ristiniemi J, Linblad I, Schiller L. Perceptions of death among children in Sweden. Int J Child Spirit. 2019;24(4):415–33. 10.1080/1364436X.2019.1672627

[9] Rabatel A, Florea M-L. Re-présentations de la mort dans les médias d’information. Quest Commun. 2011;19:7–28. 10.4000/questionsdecommunication.401

[10] Le Guay D. Représentation actuelle de la mort dans nos sociétés: Les différents moyens de l’occulter. Études sur la mort. 2008;134(2):115–23.

[11] Julier-Costes M. Le paradigme du déni social de la mort à l’épreuve des séries télévisées. Mise en scène et mise en sens de la mort. Études sur la mort. 2011;139(1):145–63. 10.3917/eslm.139.0145

[12] Ernst G, Bergeron P, Laflamme D. Mort, jeux vidéo et mondes virtuels. Frontières. 2016;28(2). 10.7202/1040191ar.

[13] Graham-Wisener L, Toner P, Leonard R, Groarke JM. Validation of the Death Literacy Index and benchmarking of death literacy level in a representative UK population sample. BMC Palliative Care. Preprint. 10.1186/s12904-022-01032-0 PMC9374575 35962383

[14] Noonan K, Horsfall D, Leonard R, Rosenberg J. Developing death literacy. Prog Palliat Care. 2016;24(1):31–5. 10.1080/09699260.2015.1103498

[15] Talwar V. Talking to children about death in educational settings. In: Talwar V, Harris PL, Schleifer M, editors. Children’s understanding of death: from biological to religious conceptions. New York: Cambridge University Press; 2011. p. 98–115.

[16] Hirsch S, Audet G, Turcotte M. Vivre ensemble. Aborder les sujets sensibles avec les élèves: Centre d’intervention pédagogique en contexte de diversité. Commission scolaire Marguerite-Bourgeois; 2015. Available from: https://www.cipcd.ca/wp-content/uploads/2014/04/CSMB_-Guide_sujets-sensibles_final..pdf.

[17] Martino W. Supporting transgender students and gender-expansive education in schools: investigating policy, pedagogy, and curricular implications. Teach Coll Rec. 2022;124(8):3–16. 10.1177/01614681221121513

[18] Papazian-Zohrabian G, Mamprin C, Lemire V, Turpin-Samson A. Prendre en compte l’expérience pré-, péri- et post-migratoire des élèves réfugiés afin de favoriser leur accueil et leur expérience socioscolaire. Alterstice. 2018;8(2):101–16. 10.7202/1066956ar

[19] Levac D, Colquhoun H, O’Brien KK. Scoping studies: advancing the methodology. Implement Sci. 2010;5(1):69. 20854677 10.1186/1748-5908-5-69 PMC2954944

[20] Knowledge and practices of youth awareness on death and dying in school settings: protocol registration. Open Science Framework. 2023. 10.17605/OSF.IO/EHY8T.

[21] Peters M, Godfrey C, McInerney P, Munn Z, Tricco A, Khalil H. Scoping reviews. In: Aromataris E, Munn Z, editors. JBI Manual for evidence synthesis. Joanna Briggs Institute; 2020. 10.46658/JBIRM-19-01.

[22] Kellehear A. On dying and human suffering. Palliat Med. 2009;23(5):388–97. 19477888 10.1177/0269216309104858

[23] Chauhan S. A meta-analysis of the impact of technology on learning effectiveness of elementary students. Comput Educ. 2017;105:14–30. 10.1016/j.compedu.2016.11.005

[24] Rahmatullah AS, Mulyasa E, Syahrani S, Pongpalilu F, Putri RE. Digital era 40: the contribution to education and student psychology. Linguist Cult Rev. 2022;6(S3):89–107. 10.21744/lingcure.v6nS3.2064

[25] Hashim H. Application of technology in the digital era education. Int J Res Couns Educ. 2018;2(1):1.

[26] Tricco AC, Lillie E, Zarin W, O’Brien KK, Colquhoun H, Levac D, et al. PRISMA Extension for Scoping Reviews (PRISMA-ScR): checklist and explanation. Ann Intern Med. 2018;169(7):467–73. 30178033 10.7326/M18-0850

[27] Miles M, Huberman M, Saldana J. Qualitative data analysis: a sourcebook of new methods. 3rd ed. Beverly Hills: Sage Publications; 2014. p. 263.

