# Supplementary material for: Knowledge and practices of youth awareness on death and dying in school settings: a systematic scoping review protocol
Source: Syst Rev. 2024 Aug 24;13:220. doi: 10.1186/s13643-024-02635-9 (PMC11344924; doi:10.1186/s13643-024-02635-9)
Supplement: Supplementary file 2 — Supplementary Material 2: Database. [file 13643_2024_2635_MOESM2_ESM.docx]

# **Design of research concepts and equations**

## Databases to use:

- CINAHL Complete (EBSCO)
- MEDLINE (Ovid)
- EBM Reviews_Cochrane(Ovid)
- JBI EBP Database (Ovid)
- PsycINFO (Ovid)
- W of S (Clarivate)
- Global health (OVID)
- Sociological Abstracts (ProQuest)
- Social Sciences Abstracts (EBSCO)
- Family Studies Abstracts (EBSCO)
- Social Services Abstracts (ProQuest)
- Social Work Abstracts (EBSCO)
- Erudit (Francophone)
- CAIRN (Francophone)
- PubPsy

## Grey literature:

- Dissertations & Theses Global (ProQuest)
- Google Scholar

## Search strategy in CINAHL Complete (EBSCO)

| **Concepts** | **Awareness / education about dying and death** | **Young people** | **School** |
| --- | --- | --- | --- |
| **Thesaurus CINAHL** | (MH "Death Education")  (MH "Attitude to Death")  (MH "Death+")  (MH "Terminal Care+")  (MH "Bereavement+")  (MH "Suicide, Assisted")  (MH "Terminally Ill Patients")  (MH "Hospice Patients")  (MH "Hospices")  (MH "Burial Practices") | (MH "Child")  (MH "Adolescence") | (MH "Schools, Middle")  (MH "Schools, Elementary")  (MH "Schools, Secondary")  (MH "Schools, Special")  (MH "Teachers")  (MH "Curriculum+")  (MH "Students, High School")  (MH "Students, Middle School")  (MH "Students, Elementary") |

| **Concept 1 : thesaurus strategy (S1)** | ((MH "Death Education") OR (MH "Attitude to Death") OR (MH "Death+") OR (MH "Terminal Care+") OR (MH "Bereavement+") OR (MH "Terminally Ill Patients") OR (MH "Hospice Patients") OR (MH "Hospices") OR (MH "Suicide, Assisted") OR (MH "Burial Practices")) | |
| --- | --- | --- |
| **Concept 1 : keywords strategy (S2)** | TI ( Death* OR Dying OR Palliati* OR Hospice* OR Euthanasia OR Bereav* OR bereft OR grief OR grieving OR mourning OR funeral* ) OR AB ( Death* OR Dying OR Palliati* OR Hospice* OR Euthanasia OR Bereav* OR bereft OR grief OR grieving OR mourning OR funeral* ) OR MW ( Death* OR Dying OR Palliati* OR Hospice* OR Euthanasia OR Bereav* OR bereft OR grief OR grieving OR mourning OR funeral* ) OR TI ( (Terminal* N1 (care OR ill*)) OR (suicide N2 assist*) ) OR AB ( (Terminal* N1 (care OR ill*)) OR (suicide N2 assist*) ) OR MW ( (Terminal* N1 (care OR ill*)) OR (suicide N2 assist*) )  OR TI ( "End-of life" OR "Supportive care" ) OR AB ( "End-of life" OR "Supportive care" ) OR MW ( "End-of life" OR "Supportive care" ) | |
| **Concept 1 : mixed strategy (S3)** | **OR** | **(S1 OR S2)** |
| **Concept 2 : thesaurus strategy (S4)** | (MH "Child") OR (MH "Adolescence") | |
| **Concept 2 : keywords strategy (S5)** | TI ( Youth* OR Child* OR Boy* OR Girl* OR Kid OR Kids OR Adolescen* OR Teen* ) OR AB ( Youth* OR Child* OR Boy* OR Girl* OR Kid OR Kids OR Adolescen* OR Teen* ) OR MW ( Youth* OR Child* OR Boy* OR Girl* OR Kid OR Kids OR Adolescen* OR Teen* ) | |
| **Concept 2 : mixed strategy (S6)** | **OR** | **(S4 OR S5)** |
| **Concept 3 : thesaurus strategy (S7)** | (MH "Schools, Middle") OR (MH "Schools, Elementary") OR (MH "Schools, Secondary") OR (MH "Schools, Special") OR (MH "Teachers") OR (MH "Curriculum+") OR (MH "Students, High School") OR (MH "Students, Middle School") OR (MH "Students, Elementary") | |
| **Concept 3 : keywords strategy (S8)** | TI ( School* OR Kindergarten* OR Curriculum* OR Teacher* OR Pupil* ) OR AB ( School* OR Kindergarten* OR Curriculum* OR Teacher* OR Pupil* ) OR MW ( School* OR Kindergarten* OR Curriculum* OR Teacher* OR Pupil* ) OR TI ( (Education OR Student*) N1 (Primary OR Secondary OR Elementary) ) OR AB ((Education OR Student*) N1 (Primary OR Secondary OR Elementary) ) OR MW ( (Education OR Student*) N1 (Primary OR Secondary OR Elementary) ) | |
| **Concept 3 : mixed strategy (S9)** | **OR** | **(S7 OR S8)** |
| **Final strategy** | **AND** | **(S3 AND S6 AND S9)** |

## Search strategy in Medline (OVID)

| **Concepts** | **Awareness / education about dying and death** | **Young people** | **School** |
| --- | --- | --- | --- |
| **MeSH** | exp Death/  Palliative care/ or Terminal care/  bereavement/ or grief/ exp Hospice Care/  exp Hospices/ exp Euthanasia/ Suicide, Assisted/  Attitude to Death/ or Funeral Rites/ | Child/  Adolescent/ | Schools/  Students/  School Teachers/  Teaching/ exp Curriculum/ |

| **Concept 1 : thesaurus strategy (S1)** | exp Death/ or Palliative care/ or Terminal care/ or bereavement/ or grief/ or exp Hospice Care/ or exp Hospices/ or exp Euthanasia/ or Suicide, Assisted/ or Attitude to Death/ or Funeral Rites/ | |
| --- | --- | --- |
| **Concept 1 : keywords strategy (S2)** | (Death* or Dying or Palliati* or Hospice* or Euthanasia or Bereav* or Bereft or Grief or Grieving or Mourning or Funeral*).ab,kf,ti.  ((Terminal* adj1 (care OR ill*)) OR (suicide adj2 assist*)).ab,kf,ti.  ("End of life" OR "Supportive care").ab,kf,ti. | |
| **Concept 1 : mixed strategy (S3)** | **OR** | **(S1 OR S2)** |
| **Concept 2 : thesaurus strategy (S4)** | Child/ or Adolescent/ | |
| **Concept 2 : keywords strategy (S5)** | (Youth* or Child* or Boy* or Girl* or Kid or Kids or Adolescen* or Teen*).ab,kf,ti. | |
| **Concept 2 : mixed strategy (S6)** | **OR** | **(S4 OR S5)** |
| **Concept 3 : thesaurus strategy (S7)** | Schools/ or Students/ or School Teachers/ or Teaching/ or exp Curriculum/ | |
| **Concept 3 : keywords strategy (S8)** | (School* or Kindergarten* or Curriculum* or Teacher* or Pupil*).ab,kf,ti.  ((Education OR Student*) adj1 (Primary OR Secondary OR Elementary)).ab,kf,ti. | |
| **Concept 3 : mixed strategy (S9)** | **OR** | **(S7 OR S8)** |
| **Final strategy** | **AND** | **(S3 AND S6 AND S9)** |

## Search strategy in PsycInfo (OVID)

| **Concepts** | **Awareness / education about dying and death** | **Young people** | | **School** | | |
| --- | --- | --- | --- | --- | --- | --- |
| **Thesaurus** | "death and dying"/  assisted suicide/  child death/  euthanasia/  parental death/  partner death/  exp sudden death/ Death Education/  exp Bereavement/  exp Palliative care/  Advance Directives/  terminally ill patients/  hospice/  terminal cancer/  Death attitudes/  Death Rites/ | **school age 6 12 yrs.ag.**  **adolescence 13 17 yrs.ag.** | | Junior High Schools/  High Schools/  Middle Schools/  Elementary Schools/  kindergartens/  elementary school teachers/  high school teachers/  junior high school teachers/  middle school teachers/  special education teachers/ curriculum/  exp elementary school students/  exp high school graduates/  exp high school students/  exp junior high school students/  exp kindergarten students/  exp middle school students/ | | |
| **Concept 1 : thesaurus strategy (S1)** | | | "death and dying"/ or assisted suicide/ or child death/ or euthanasia/ or parental death/ or partner death/ or exp sudden death/ or Death Education/ or exp Bereavement/ or exp Palliative care/ or Advance Directives/ or terminally ill patients/ or hospice/ or terminal cancer/ or Death attitudes/ or Death Rites/ | | |  |
| **Concept 1 : keywords strategy (S2)** | | | (Death* OR Dying OR Palliati* OR Hospice* OR Euthanasia OR Bereav* OR Bereft OR Grief OR Grieving OR Mourning OR Funeral*).ab,id,ti. OR ((Terminal* adj1 (care OR ill*)) OR (suicide adj2 assist*)).ab,id,ti. OR ("End of life" OR "Supportive care").ab,id,ti. | | |  |
| **Concept 1 : mixed strategy (S3)** | | | **OR** | | **(S1 OR S2)** |  |
| **Concept 2 : thesaurus strategy (S4)** | | | **school age 6 12 yrs.ag. or adolescence 13 17 yrs.ag.** | | |  |
| **Concept 2 : keywords strategy (S5)** | | | (Youth* or Child* or Boy* or Girl* or Kid or Kids or Adolescen* or Teen*).ab,id,ti. | | |  |
| **Concept 2 : mixed strategy (S6)** | | | **OR** | | **(S4 OR S5)** |  |
| **Concept 3 : thesaurus strategy (S7)** | | | Junior High Schools/ or High Schools/ or Middle Schools/ or Elementary Schools/ or kindergartens/ or elementary school teachers/ or high school teachers/ or junior high school teachers/ or middle school teachers/ or special education teachers/ or curriculum/ or exp elementary school students/ or exp high school graduates/ or exp high school students/ or exp junior high school students/ or exp kindergarten students/ or exp middle school students/ | | |  |
| **Concept 3 : keywords strategy (S8)** | | | (School* or Kindergarten* or Curriculum* or Teacher* or Pupil*).ab,id,ti.  OR ((Education OR Student*) adj1 (Primary OR Secondary OR Elementary)).ab,id,ti. | | |  |
| **Concept 3 : mixed strategy (S9)** | | | **OR** | | **(S7 OR S8)** |  |
| **Final strategy** | | | **AND** | | **(S3 AND S6 AND S9)** |  |

## Search strategy in EBM Reviews - Cochrane Database of systematics reviews (OVID)

| **Concept 1 : keywords strategy (S1)** | (Death* or Dying or Palliati* or Hospice* or Euthanasia or Bereav* or Bereft or Grief or Grieving or Mourning or Funeral*).ti,ab,kw. or ((Terminal* adj1 (care OR ill*)) OR (suicide adj2 assist*)).ti,ab,kw. or ("End of life" or "Supportive care").ti,ab,kw. |
| --- | --- |
| **Concept 2 : keywords strategy (S2)** | (Youth* or Child* or Boy* or Girl* or Kid or Kids or Adolescen* or Teen*). ti,ab,kw. |
| **Concept 3 : keywords strategy (S3)** | (School* or Kindergarten* or Curriculum* or Teacher* or Pupil*).ti,ab,kw. or ((Education OR Student*) adj1 (Primary OR Secondary OR Elementary)).ti,ab,kw. |
| **Final strategy** | **(S1 AND S2 AND S3)** |

## Search strategy in JBI EBP Database (OVID)

| **Concept 1 : keywords strategy (S1)** | (Death* or Dying or Palliati* or Hospice* or Euthanasia or Bereav* or Bereft or Grief or Grieving or Mourning or Funeral*).ti,ab,hw.  ((Terminal* adj1 (care OR ill*)) OR (suicide adj2 assist*)).ti,ab,hw.  ("End of life" or "Supportive care").ti,ab,hw. |
| --- | --- |
| **Concept 2 : keywords strategy (S2)** | (Youth* or Child* or Boy* or Girl* or Kid or Kids or Adolescen* or Teen*).ti,ab,hw. |
| **Concept 3 : keywords strategy (S3)** | (School* or Kindergarten* or Curriculum* or Teacher* or Pupil*).ti,ab,hw.  ((Education OR Student*) adj1 (Primary OR Secondary OR Elementary)).ti,ab,hw. |
| **Final strategy** | **(S3 AND S6 AND S9)** |

## Search strategy in Global Health (OVID)

| **Concepts** | **Awareness / education about dying and death** | **Young people** | **School** |
| --- | --- | --- | --- |
| **Thesaurus** | "death and dying"/  exp death/ hospices/  palliative care/ euthanasia/  hospice care/ | adolescents/  exp youth/  adolescence/  children/  boys/  girls/  school children/ | kindergarten/  elementary schools/  high schools/  curriculum/  primary education/  school children/  secondary education/  teachers/ |

| **Concept 1 : thesaurus strategy (S1)** | "death and dying"/ or exp death/ or hospices/ or palliative care/ or euthanasia/ or hospice care/ | |
| --- | --- | --- |
| **Concept 1 : keywords strategy (S2)** | (Death* or Dying or Palliati* or Hospice* or Euthanasia or Bereav* or Bereft or Grief or Grieving or Mourning or Funeral*).ab,hw,ti.  ((Terminal* adj1 (care OR ill*)) OR (suicide adj2 assist*)).ab,hw,ti.  ("End of life" or "Supportive care").ab,hw,ti. | |
| **Concept 1 : mixed strategy (S3)** | **OR** | **(S1 OR S2)** |
| **Concept 2 : thesaurus strategy (S4)** | adolescents/ or exp youth/ or adolescence/ or children/ or boys/ or girls/ or school children/ | |
| **Concept 2 : keywords strategy (S5)** | (Youth* or Child* or Boy* or Girl* or Kid or Kids or Adolescen* or Teen*).ab,hw,ti. | |
| **Concept 2 : mixed strategy (S6)** | **OR** | **(S4 OR S5)** |
| **Concept 3 : thesaurus strategy (S7)** | kindergarten/ or elementary schools/ or high schools/ or curriculum/ or primary education/ or school children/ or secondary education/ or teachers/ | |
| **Concept 3 : keywords strategy (S8)** | (School* or Kindergarten* or Curriculum* or Teacher* or Pupil*).ab,hw,ti.  ((Education OR Student*) adj1 (Primary OR Secondary OR Elementary)).ab,hw,ti. | |
| **Concept 3 : mixed strategy (S9)** | **OR** | **(S7 OR S8)** |
| **Final strategy** | **(S3 AND S6 AND S9)** | |

## Search strategy in Web of Science

| **Concept 1 : keywords strategy (S1)** | **((TS=((Death* OR Dying OR Palliati* OR Hospice* OR Euthanasia OR Bereav* OR Bereft OR Grief OR Grieving OR Mourning OR Funeral*) )) OR TS=(((Terminal* NEAR/1 (care OR ill*)) OR (suicide NEAR/1 assist*)) )) OR TS=(("End of life" or "Supportive care") )** |
| --- | --- |
| **Concept 2 : keywords strategy (S2)** | **TS=((Youth* OR Child* OR Boy* OR Girl* OR Kid OR Kids OR Adolescen* OR Teen*) )** |
| **Concept 3 : keywords strategy (S3)** | **(TS=((School* OR Kindergarten* OR Curriculum* OR Teacher* OR Pupil*) )) OR TS=(((Education OR Student*) NEAR/1 (Primary OR Secondary OR Elementary)) )** |
| **Final strategy** | **(S1 AND S2 AND S3)** |

## Search strategy in Social Sciences Abstracts (EBSCO)

| **Concepts** | **Awareness / education about dying and death** | **Young people** | **School** |
| --- | --- | --- | --- |
| **Thesaurus** | "Death"  "Children & death"  "Right to die"  "Teenagers & death"  "Youth & death"  "Bereavement"  "Grief"  "Attitudes toward death"  "Hospices Terminal care facilities"  "Terminal care"  "Hospice care"  "Terminally ill"  "Terminal care facilities"  "Euthanasia"  "Passive euthanasia"  "Assisted suicide"  "Mourning customs"  "Funerals" | "School children"  "Teenage girls"  "Teenage boys"  "Adolescence"  "Teenagers"  "Children" | "Elementary school teachers"  "High school teachers"  "Elementary school teachers"  "High school teachers"  "Kindergarten teachers"  "High schools"  "Suburban high schools"  "Urban high schools"  "Middle school students"  "Junior high schools"  "Junior high school students"  "Middle schools"  "Secondary education"  "Elementary schools"  "Kindergarten"  "Primary education"  "Elementary education"  "Curriculum" |

| **Concept 1 : thesaurus strategy (S1)** | (DE "Death" OR DE "Children & death" OR DE "Right to die" OR DE "Teenagers & death" OR DE "Youth & death" OR DE "Bereavement" OR DE "Grief" OR DE "Attitudes toward death" OR DE "Hospices Terminal care facilities" OR DE "Terminal care" OR DE "Hospice care" OR DE "Terminally ill" OR DE "Terminal care facilities" OR DE "Euthanasia" OR DE "Passive euthanasia" OR DE "Assisted suicide" OR DE "Mourning customs" OR DE "Funerals") | |
| --- | --- | --- |
| **Concept 1 : keywords strategy (S2)** | TI ( Death* or Dying or Palliati* or Hospice* or Euthanasia or Bereav* or Bereft Grief or Grieving or Mourning OR Funeral* ) OR AB ( Death* or Dying or Palliati* or Hospice* or Euthanasia or Bereav* or Bereft or Grief or Grieving or Mourning OR Funeral* ) OR SU ( Death* or Dying or Palliati* or Hospice* or Euthanasia or Bereav* or Bereft or Grief or Grieving or Mourning OR Funeral* )  TI ( (Terminal* N1 (Care or Ill*)) OR (suicide N1 assist*) ) OR AB ( (Terminal* N1 (Care or Ill*)) OR (suicide N1 assist*) ) OR SU ( (Terminal* N1 (Care or Ill*) OR (suicide N1 assist*)) )  TI ( "End of life" or "Supportive care" ) OR AB ( "End of life" or "Supportive care" ) OR SU ( "End of life" or "Supportive care" ) | |
| **Concept 1 : mixed strategy (S3)** | **OR** | **(S1 OR S2)** |
| **Concept 2 : thesaurus strategy (S4)** | (DE "School children" OR DE "Teenage girls" OR DE "Teenage boys" OR DE "Adolescence" OR DE "Teenagers" OR DE "Children") | |
| **Concept 2 : keywords strategy (S5)** | TI ( (Youth* or Child* or Boy* or Girl* or Kid or Kids or Adolescen* or Teen*) ) OR AB ( (Youth* or Child* or Boy* or Girl* or Kid or Kids or Adolescen* or Teen*) ) OR SU ( (Youth* or Child* or Boy* or Girl* or Kid or Kids or Adolescen* or Teen*) ) | |
| **Concept 2 : mixed strategy (S6)** | **OR** | **(S4 OR S5)** |
| **Concept 3 : thesaurus strategy (S7)** | (DE "Elementary school teachers" OR DE "High school teachers" DE "Elementary school teachers" OR DE "High school teachers" OR DE "Kindergarten teachers" OR DE "High schools" OR DE "Suburban high schools" OR DE "Urban high schools" OR DE "Middle school students" OR DE "Junior high schools" OR DE "Junior high school students" OR DE "Middle schools" OR DE "Secondary education" OR DE "Elementary schools" OR DE "Kindergarten" OR DE "Primary education" OR DE "Elementary education" OR DE "Curriculum") | |
| **Concept 3 : keywords strategy (S8)** | TI ( School* or Kindergarten* or Curriculum* or Teacher* or Pupil* ) OR AB ( School* or Kindergarten* or Curriculum* or Teacher* or Pupil* ) OR SU ( School* or Kindergarten* or Curriculum* or Teacher* or Pupil* )  TI ( (Education OR Student*) N1 (Primary OR Secondary OR Elementary) ) OR AB ( (Education OR Student*) N1 (Primary OR Secondary OR Elementary) ) OR SU ( (Education OR Student*) N1 (Primary OR Secondary OR Elementary) ) | |
| **Concept 3 : mixed strategy (S9)** | **OR** | **(S7 OR S8)** |
| **Final strategy** | **(S3 AND S6 AND S9)** | |

## Search strategy in Families Studies Abstracts (EBSCO)

| **Concept 1 : keywords strategy (S1)** | TI ( Death* or Dying or Palliati* or Hospice* or Euthanasia or Bereav* or Bereft Grief or Grieving or Mourning OR Funeral* ) OR AB ( Death* or Dying or Palliati* or Hospice* or Euthanasia or Bereav* or Bereft or Grief or Grieving or Mourning OR Funeral* ) OR SU ( Death* or Dying or Palliati* or Hospice* or Euthanasia or Bereav* or Bereft or Grief or Grieving or Mourning ) OR KW ( Death* or Dying or Palliati* or Hospice* or Euthanasia or Bereav* or Bereft or Grief or Grieving or Mourning OR Funeral* )  TI ( (Terminal* N1 (Care or Ill*)) OR (suicide N1 assist*) ) OR AB ( (Terminal* N1 (Care or Ill*)) OR (suicide N1 assist*) ) OR SU ( (Terminal* N1 (Care or Ill*)) OR (suicide N1 assist*) ) OR KW ( (Terminal* N1 (Care or Ill*)) OR (suicide N1 assist*) )  TI ( "End of life" or "Supportive care" ) OR AB ( "End of life" or "Supportive care" ) OR SU ( "End of life" or "Supportive care" ) OR KW ( "End of life" or "Supportive care" ) |
| --- | --- |
| **Concept 2 : keywords strategy (S2)** | TI ( (Youth* or Child* or Boy* or Girl* or Kid or Kids or Adolescen* or Teen*) ) OR SU ( (Youth* or Child* or Boy* or Girl* or Kid or Kids or Adolescen* or Teen*) ) OR AB ( (Youth* or Child* or Boy* or Girl* or Kid or Kids or Adolescen* or Teen*) ) OR KW ( (Youth* or Child* or Boy* or Girl* or Kid or Kids or Adolescen* or Teen*) ) |
| **Concept 3 : keywords strategy (S3)** | TI ( School* or Kindergarten* or Curriculum* or Teacher* or Pupil* ) OR AB ( School* or Kindergarten* or Curriculum* or Teacher* or Pupil* ) OR SU ( School* or Kindergarten* or Curriculum* or Teacher* or Pupil* ) OR KW ( School* or Kindergarten* or Curriculum* or Teacher* or Pupil* )  TI ( (Education OR Student*) N1 (Primary OR Secondary OR Elementary) ) OR AB ( (Education OR Student*) N1 (Primary OR Secondary OR Elementary) ) OR SU ( (Education OR Student*) N1 (Primary OR Secondary OR Elementary) ) OR KW ( (Education OR Student*) N1 (Primary OR Secondary OR Elementary) ) |
| **Final strategy** | **(S1 AND S2 AND S3)** |

## Search strategy in Social Work Abstracts (EBSCO)

| **Concept 1 : keywords strategy (S1)** | TI ( Death* or Dying or Palliati* or Hospice* or Euthanasia or Bereav* or Bereft Grief or Grieving or Mourning OR Funeral* ) OR AB ( Death* or Dying or Palliati* or Hospice* or Euthanasia or Bereav* or Bereft or Grief or Grieving or Mourning OR Funeral* ) OR SU ( Death* or Dying or Palliati* or Hospice* or Euthanasia or Bereav* or Bereft or Grief or Grieving or Mourning ) OR KW ( Death* or Dying or Palliati* or Hospice* or Euthanasia or Bereav* or Bereft or Grief or Grieving or Mourning OR Funeral* )  TI ( (Terminal* N1 (Care or Ill*)) OR (suicide N1 assist*) ) OR AB ( (Terminal* N1 (Care or Ill*)) OR (suicide N1 assist*) ) OR SU ( (Terminal* N1 (Care or Ill*)) OR (suicide N1 assist*) ) OR KW ( (Terminal* N1 (Care or Ill*)) OR (suicide N1 assist*) )  TI ( "End of life" or "Supportive care" ) OR AB ( "End of life" or "Supportive care" ) OR SU ( "End of life" or "Supportive care" ) OR KW ( "End of life" or "Supportive care" ) |
| --- | --- |
| **Concept 2 : keywords strategy (S2)** | TI ( (Youth* or Child* or Boy* or Girl* or Kid or Kids or Adolescen* or Teen*) ) OR SU ( (Youth* or Child* or Boy* or Girl* or Kid or Kids or Adolescen* or Teen*) ) OR AB ( (Youth* or Child* or Boy* or Girl* or Kid or Kids or Adolescen* or Teen*) ) OR KW ( (Youth* or Child* or Boy* or Girl* or Kid or Kids or Adolescen* or Teen*) ) |
| **Concept 3 : keywords strategy (S3)** | TI ( School* or Kindergarten* or Curriculum* or Teacher* or Pupil* ) OR AB ( School* or Kindergarten* or Curriculum* or Teacher* or Pupil* ) OR SU ( School* or Kindergarten* or Curriculum* or Teacher* or Pupil* ) OR KW ( School* or Kindergarten* or Curriculum* or Teacher* or Pupil* )  TI ( (Education OR Student*) N1 (Primary OR Secondary OR Elementary) ) OR AB ( (Education OR Student*) N1 (Primary OR Secondary OR Elementary) ) OR SU ( (Education OR Student*) N1 (Primary OR Secondary OR Elementary) ) OR KW ( (Education OR Student*) N1 (Primary OR Secondary OR Elementary) ) |
| **Final strategy** | **(S1 AND S2 AND S3)** |

## Search strategy in Sociological Abstracts (ProQuest)

| **Concepts** | **Awareness / education about dying and death** | **Young people** | **School** |
| --- | --- | --- | --- |
| **Thesaurus** | "Death Attitudes"  "Grief"  "Terminal Illness"  "Death"  "Dying"  "Euthanasia"  "Hospices"  "Palliative Care"  EXPLODE "Death Rituals"  "Right to Die" | "Children"  "Adolescents"  "Youth" | "Curriculum"  "Teachers"  "Junior High School Students"  "High School Students"  "Elementary School Students"  EXPLODE "Elementary Education"  EXPLODE "Secondary Schools"  "Secondary Education"  "Elementary Schools"  "Kindergarten" |

| **Concept 1 : thesaurus strategy (S1)** | MAINSUBJECT.EXACT("Death Attitudes") OR MAINSUBJECT.EXACT("Grief") OR MAINSUBJECT.EXACT("Terminal Illness") OR MAINSUBJECT.EXACT("Death") OR MAINSUBJECT.EXACT("Dying") OR MAINSUBJECT.EXACT("Euthanasia") OR MAINSUBJECT.EXACT("Hospices") OR MAINSUBJECT.EXACT("Palliative Care")  OR MAINSUBJECT.EXACT.EXPLODE("Death Rituals") OR MAINSUBJECT.EXACT("Right to Die") | |
| --- | --- | --- |
| **Concept 1 : keywords strategy (S2)** | title(Death* OR Dying OR Palliati* OR Hospice* OR Euthanasia OR Bereav* OR Bereft OR Grief OR Grieving OR Mourning OR Funeral*) OR abstract(Death* OR Dying OR Palliati* OR Hospice* OR Euthanasia OR Bereav* OR Bereft OR Grief OR Grieving OR Mourning OR Funeral*) OR subject(Death* OR Dying OR Palliati* OR Hospice* OR Euthanasia OR Bereav* OR Bereft OR Grief OR Grieving OR Mourning OR Funeral*) OR Title((Terminal* NEAR/1 (Care OR Ill*)) OR (suicide NEAR/1 assist*)) OR abstract ((Terminal* NEAR/1 (Care OR Ill*)) OR (suicide NEAR/1 assist*))) OR subject OR ((Terminal* NEAR/1 (Care OR Ill*)) OR (suicide NEAR/1 assist*))) OR Title("End of life" OR "Supportive care") OR abstract("End of life" OR "Supportive care") OR subject("End of life" OR "Supportive care") | |
| **Concept 1 : mixed strategy (S3)** | **OR** | **(S1 OR S2)** |
| **Concept 2 : thesaurus strategy (S4)** | (MAINSUBJECT.EXACT("Children") OR MAINSUBJECT.EXACT("Adolescents") OR MAINSUBJECT.EXACT("Youth")) | |
| **Concept 2 : keywords strategy (S5)** | title((Youth* OR Child* OR Boy* OR Girl* OR Kid OR Kids OR Adolescen* OR Teen*)) OR abstract((Youth* OR Child* OR Boy* OR Girl* OR Kid OR Kids OR Adolescen* OR Teen*)) OR subject((Youth* OR Child* OR Boy* OR Girl* OR Kid OR Kids OR Adolescen* OR Teen*)) | |
| **Concept 2 : mixed strategy (S6)** | **OR** | **(S4 OR S5)** |
| **Concept 3 : thesaurus strategy (S7)** | MAINSUBJECT.EXACT("Curriculum") OR MAINSUBJECT.EXACT("Teachers") OR MAINSUBJECT.EXACT("Junior High School Students") OR MAINSUBJECT.EXACT("High School Students") OR MAINSUBJECT.EXACT("Elementary School Students") OR MAINSUBJECT.EXACT.EXPLODE("Elementary Education") OR MAINSUBJECT.EXACT.EXPLODE("Secondary Schools") OR MAINSUBJECT.EXACT("Secondary Education") OR MAINSUBJECT.EXACT("Elementary Schools") OR MAINSUBJECT.EXACT("Kindergarten") | |
| **Concept 3 : keywords strategy (S8)** | title(School* or Kindergarten* or Curriculum* or Teacher* or Pupil*) OR abstract(School* or Kindergarten* or Curriculum* or Teacher* or Pupil*) OR subject(School* or Kindergarten* or Curriculum* or Teacher* or Pupil*) OR title((Education OR Student*) NEAR/1 (Primary OR Secondary OR Elementary)) OR abstract((Education OR Student*) NEAR/1 (Primary OR Secondary OR Elementary)) OR subject((Education OR Student*) NEAR/1 (Primary OR Secondary OR Elementary)) | |
| **Concept 3 : mixed strategy (S9)** | **OR** | **(S7 OR S8)** |
| **Final strategy** | **AND** | **(S3 AND S6 AND S9)** |

## Search strategy in Social Services Abstracts (ProQuest)

| **Concepts** | **Awareness / education about dying and death** | **Young people** | **School** |
| --- | --- | --- | --- |
| **Thesaurus** | "Death Attitudes"  "Grief"  "Terminal Illness"  "Death"  "Dying"  "Euthanasia"  "Hospices"  "Palliative Care"  "Death Rituals" (EXPLODE)  "Right to Die" | "Children"  "Adolescents"  "Youth" | "Curriculum"  "Teachers"  "Junior High School Students"  "High School Students"  "Elementary School Students" EXPLODE("Elementary Education") EXPLODE("Secondary Schools")  "Secondary Education"  "Elementary Schools"  "Kindergarten" |

| **Concept 1 : thesaurus strategy (S1)** | MAINSUBJECT.EXACT("Death Attitudes") OR MAINSUBJECT.EXACT("Grief") OR MAINSUBJECT.EXACT("Terminal Illness") OR MAINSUBJECT.EXACT("Death") OR MAINSUBJECT.EXACT("Dying") OR MAINSUBJECT.EXACT("Euthanasia") OR MAINSUBJECT.EXACT("Hospices") OR MAINSUBJECT.EXACT("Palliative Care")  OR MAINSUBJECT.EXACT.EXPLODE("Death Rituals") OR MAINSUBJECT.EXACT("Right to Die") | |
| --- | --- | --- |
| **Concept 1 : keywords strategy (S2)** | title(Death* OR Dying OR Palliati* OR Hospice* OR Euthanasia OR Bereav* OR Bereft OR Grief OR Grieving OR Mourning OR Funeral*) OR abstract(Death* OR Dying OR Palliati* OR Hospice* OR Euthanasia OR Bereav* OR Bereft OR Grief OR Grieving OR Mourning OR Funeral*) OR subject(Death* OR Dying OR Palliati* OR Hospice* OR Euthanasia OR Bereav* OR Bereft OR Grief OR Grieving OR Mourning OR Funeral*) OR Title(((Terminal* NEAR/1 (Care OR Ill*)) OR (suicide NEAR/1 assist*))) OR abstract(((Terminal* NEAR/1 (Care OR Ill*)) OR (suicide NEAR/1 assist*))) OR subject (((Terminal* NEAR/1 (Care OR Ill*)) OR (suicide NEAR/1 assist*))) OR title("End of life" OR "Supportive care") OR abstract("End of life" OR "Supportive care") OR subject("End of life" OR "Supportive care") | |
| **Concept 1 : mixed strategy (S3)** | **OR** | **(S1 OR S2)** |
| **Concept 2 : thesaurus strategy (S4)** | MAINSUBJECT.EXACT("Children") OR MAINSUBJECT.EXACT("Adolescents") OR MAINSUBJECT.EXACT("Youth") | |
| **Concept 2 : keywords strategy (S5)** | title((Youth* OR Child* OR Boy* OR Girl* OR Kid OR Kids OR Adolescen* OR Teen*)) OR abstract((Youth* OR Child* OR Boy* OR Girl* OR Kid OR Kids OR Adolescen* OR Teen*)) OR subject((Youth* OR Child* OR Boy* OR Girl* OR Kid OR Kids OR Adolescen* OR Teen*)) | |
| **Concept 2 : mixed strategy (S6)** | **OR** | **(S4 OR S5)** |
| **Concept 3 : thesaurus strategy (S7)** | MAINSUBJECT.EXACT("Curriculum") OR MAINSUBJECT.EXACT("Teachers") OR MAINSUBJECT.EXACT("Junior High School Students") OR MAINSUBJECT.EXACT("High School Students") OR MAINSUBJECT.EXACT("Elementary School Students") OR MAINSUBJECT.EXACT.EXPLODE("Elementary Education") OR MAINSUBJECT.EXACT.EXPLODE("Secondary Schools") OR MAINSUBJECT.EXACT("Secondary Education") OR MAINSUBJECT.EXACT("Elementary Schools") OR MAINSUBJECT.EXACT("Kindergarten") | |
| **Concept 3 : keywords strategy (S8)** | title(School* or Kindergarten* or Curriculum* or Teacher* or Pupil*) OR abstract(School* or Kindergarten* or Curriculum* or Teacher* or Pupil*) OR subject(School* or Kindergarten* or Curriculum* or Teacher* or Pupil*) OR title((Education OR Student*) NEAR/1 (Primary OR Secondary OR Elementary)) OR abstract((Education OR Student*) NEAR/1 (Primary OR Secondary OR Elementary)) OR subject((Education OR Student*) NEAR/1 (Primary OR Secondary OR Elementary)) | |
| **Concept 3 : mixed strategy (S9)** | **OR** | **(S7 OR S8)** |
| **Final strategy** | **AND** | **(S3 AND S6 AND S9)** |

## Search strategy in Érudit ('Érudit', 'UNB', 'Persée', 'FRQ')

### English search

| **Concept 1 : keywords strategy (S1)** | (Titre, résumé, mots-clés : (Death* OR Dying OR Palliati* OR Hospice* OR Euthanasia OR Bereav* OR Bereft OR Grief OR Grieving OR Mourning OR Funeral*)) OR (Titre, résumé, mots-clés : ("terminal care" OR "terminal illness" OR "terminally ill") OR (Titre, résumé, mots-clés : ("End of life" OR "Supportive care" OR "Assisted suicide" OR "Assistance in suicide")) |
| --- | --- |
| **Concept 2 : keywords strategy (S2)** | (Titre, résumé, mots-clés : (Youth* OR Child* OR Boy* OR Girl* OR Kid OR Kids OR Adolescen* OR Teen*) |
| **Concept 3 : keywords strategy (S3)** | (Titre, résumé, mots-clés : (School* or Kindergarten* or Curriculum* or Teacher* or Pupil*)) OR (Titre, résumé, mots-clés : ("Primary Education" OR "Primary Student*" OR "Secondary Education" OR "Secondary Student*" OR "Elementary Education" OR "Elementary Student*")) |
| **Final strategy** | **S1 AND S2 AND S3** |

### French search

| **Concept 1 : keywords strategy (S1)** | (Titre, résumé, mots-clés : (Mort OU Décès OU Mourir OU Deuil OU Endeuill* OU Euthanasie OU Funérailles)) OU (Titre, résumé, mots-clés : ("Fin de vie" OU "Suicide assisté" OU "Aide médicale à mourir")) OU (Titre, résumé, mots-clés : ("Soins de support" OU "Approche palliative" OU "Soins palliatifs")) |
| --- | --- |
| **Concept 2 : keywords strategy (S2)** | (Titre, résumé, mots-clés : (Enfan* OU Adolescen* OU Jeune* OU Fille* OU Garçon*)) |
| **Concept 3 : keywords strategy (S3)** | (Titre, résumé, mots-clés : (Collèg* OU Lycée* OU Curriculum* OU Enseignant* OU Institutrice* OU Instituteur* OU École* OU Écolier* OU Élève* OU Scolaire)) |
| **Final strategy** | **(S1 ET S2 ET S3)** |

## Search strategy in CAIRN

| **Concept 1 : keywords strategy (S1)** | (Mort OU Décès OU Mourir OU Deuil OU Endeuill* OU Euthanasie OU Funérailles OU "Fin de vie" OU "Suicide assisté" OU "Aide médicale à mourir" OU "Soins de support" OU "Approche palliative" OU "Soins palliatifs") |
| --- | --- |
| **Concept 2 : keywords strategy (S2)** | (Enfan* OU Adolescen* OU Jeune* OU Fille* OU Garçon*) |
| **Concept 3 : keywords strategy (S3)** | (Collèg* OU Lycée* OU Curriculum* OU Enseignant* OU Institutrice* OU Instituteur* OU École* OU Écolier* OU Élève* OU Scolaire) |
| **Final strategy** | **(S1 ET S2 ET S3)** |

## Search strategy in PubPsych

| **Concept 1 : keywords strategy (S1)** | (AB=(Death* OR Dying OR Palliati* OR Hospice* OR Euthanasia OR Bereav* OR Bereft OR Grief OR Grieving OR Mourning OR Funeral* OR "terminal care" OR "terminal illness" OR "terminally ill" OR "End of life" OR "Supportive care" OR "Assisted suicide" OR "Assistance in suicide")) OR  (TI=(Death* OR Dying OR Palliati* OR Hospice* OR Euthanasia OR Bereav* OR Bereft OR Grief OR Grieving OR Mourning OR Funeral* OR "terminal care" OR "terminal illness" OR "terminally ill" OR "End of life" OR "Supportive care" OR "Assisted suicide" OR "Assistance in suicide")) OR (SW=(Death* OR Dying OR Palliati* OR Hospice* OR Euthanasia OR Bereav* OR Bereft OR Grief OR Grieving OR Mourning OR Funeral* OR "terminal care" OR "terminal illness" OR "terminally ill" OR "End of life" OR "Supportive care" OR "Assisted suicide" OR "Assistance in suicide")) |
| --- | --- |
| **Concept 2 : keywords strategy (S2)** | (AB=(Youth* OR Child* OR Boy* OR Girl* OR Kid OR Kids OR Adolescen* OR Teen*)) OR (TI=(Youth* OR Child* OR Boy* OR Girl* OR Kid OR Kids OR Adolescen* OR Teen*)) OR (SW=(Youth* OR Child* OR Boy* OR Girl* OR Kid OR Kids OR Adolescen* OR Teen*)) |
| **Concept 3 : keywords strategy (S3)** | AB=(School* or Kindergarten* or Curriculum* or Teacher* or Pupil* OR "Primary Education" OR "Primary Student*" OR "Secondary Education" OR "Secondary Student*" OR "Elementary Education" OR "Elementary Student*") OR (TI=(School* or Kindergarten* or Curriculum* or Teacher* or Pupil* OR "Primary Education" OR "Primary Student*" OR "Secondary Education" OR "Secondary Student*" OR "Elementary Education" OR "Elementary Student*")) OR (SW=(School* or Kindergarten* or Curriculum* or Teacher* or Pupil* OR "Primary Education" OR "Primary Student*" OR "Secondary Education" OR "Secondary Student*" OR "Elementary Education" OR "Elementary Student*")) |
| **Final strategy**  *Due to the size limits of the search equations, two equations will be searched on this database and exported to Covidence and then the duplicates will be eliminated.* | ((AB: Death* OR Dying OR Palliati* OR Hospice* OR Euthanasia OR Bereav* OR Bereft OR Grief OR Grieving OR Mourning OR Funeral*) OR (TI: Death* OR Dying OR Palliati* OR Hospice* OR Euthanasia OR Bereav* OR Bereft OR Grief OR Grieving OR Mourning OR Funeral*) OR (SW: Death* OR Dying OR Palliati* OR Hospice* OR Euthanasia OR Bereav* OR Bereft OR Grief OR Grieving OR Mourning OR Funeral*)) **AND** ((AB: Youth* OR Child* OR Boy* OR Girl* OR Kid OR Kids OR Adolescen* OR Teen*) OR (TI: Youth* OR Child* OR Boy* OR Girl* OR Kid OR Kids OR Adolescen* OR Teen*) OR (SW: Youth* OR Child* OR Boy* OR Girl* OR Kid OR Kids OR Adolescen* OR Teen*)) **AND** ((AB: School* or Kindergarten* or Curriculum* or Teacher* or Pupil* OR "Primary Education" OR "Primary Student*" OR "Secondary Education" OR "Secondary Student*" OR "Elementary Education" OR "Elementary Student*") OR (TI: School* or Kindergarten* or Curriculum* or Teacher* or Pupil* OR "Primary Education" OR "Primary Student*" OR "Secondary Education" OR "Secondary Student*" OR "Elementary Education" OR "Elementary Student*") OR (SW: School* or Kindergarten* or Curriculum* or Teacher* or Pupil* OR "Primary Education" OR "Primary Student*" OR "Secondary Education" OR "Secondary Student*" OR "Elementary Education" OR "Elementary Student*"))  **OR**  ((AB: "terminal care" OR "terminal illness" OR "terminally ill" OR "End of life" OR "Supportive care" OR "Assisted suicide" OR "Assistance in suicide") OR (TI: "terminal care" OR "terminal illness" OR "terminally ill" OR "End of life" OR "Supportive care" OR "Assisted suicide" OR "Assistance in suicide") OR (SW: "terminal care" OR "terminal illness" OR "terminally ill" OR "End of life" OR "Supportive care" OR "Assisted suicide" OR "Assistance in suicide")) **AND** ((AB: Youth* OR Child* OR Boy* OR Girl* OR Kid OR Kids OR Adolescen* OR Teen*) OR (TI: Youth* OR Child* OR Boy* OR Girl* OR Kid OR Kids OR Adolescen* OR Teen*) OR (SW: Youth* OR Child* OR Boy* OR Girl* OR Kid OR Kids OR Adolescen* OR Teen*)) **AND** ((AB: School* or Kindergarten* or Curriculum* or Teacher* or Pupil* OR "Primary Education" OR "Primary Student*" OR "Secondary Education" OR "Secondary Student*" OR "Elementary Education" OR "Elementary Student*") OR (TI: School* or Kindergarten* or Curriculum* or Teacher* or Pupil* OR "Primary Education" OR "Primary Student*" OR "Secondary Education" OR "Secondary Student*" OR "Elementary Education" OR "Elementary Student*") OR (SW: School* or Kindergarten* or Curriculum* or Teacher* or Pupil* OR "Primary Education" OR "Primary Student*" OR "Secondary Education" OR "Secondary Student*" OR "Elementary Education" OR "Elementary Student*")) |

## Search strategy in Google Scholar

In Google Scholar, the equation is too long and is cut at the end of the 1st concept; you have to choose the main keywords, because in any case, Google Scholar gives more weight to the first keywords than to the following ones and the number of results it finds with the same equation is sometimes variable.

### English search

(Death OR Dying OR Palliative OR Euthanasia OR Bereavement OR Grief OR "terminal care" OR "terminal illness" OR "End of life" OR "Supportive care" OR "Assisted suicide") AND (Youth OR Children OR Adolescent OR Adolescence OR Teenager) AND (School OR OR Teacher OR "Primary Education" OR "Secondary Education" OR "Elementary Education")

### French search

(Mort OU Mourir OU Décès OU Deuil OU Euthanasie OU "Fin de vie" OU "Suicide assisté" OU "Aide médicale à mourir" OU "Soins palliatifs") ET (Enfant OU Adolescent OU Jeunes) ET (École OU Collège OU Lycée OU Enseignant OU Scolaire)

## Search strategy in Dissertations & Theses Global (ProQuest)

| **Concept 1 : thesaurus strategy (S1)** | Mainsubject.Exact("assisted suicide" OR "terminal illnesses" OR "funerals" OR "hospice care" OR "euthanasia" OR "palliative care" OR "near death experiences" OR "deaths" OR "death & dying" OR "grief") |
| --- | --- |
| **Concept 1 : keywords strategy (S2)** | abstract(Death* OR Dying OR Palliati* OR Hospice* OR Euthanasia OR Bereav* OR Bereft OR Grief OR Grieving OR Mourning OR Funeral*) OR title(Death* OR Dying OR Palliati* OR Hospice* OR Euthanasia OR Bereav* OR Bereft OR Grief OR Grieving OR Mourning OR Funeral*) OR subject(Death* OR Dying OR Palliati* OR Hospice* OR Euthanasia OR Bereav* OR Bereft OR Grief OR Grieving OR Mourning OR Funeral*)  abstract(((Terminal* NEAR/1 (Care OR Ill*)) OR (suicide NEAR/1 assist*))) OR title(((Terminal* NEAR/1 (Care OR Ill*)) OR (suicide NEAR/1 assist*))) OR subject(((Terminal* NEAR/1 (Care OR Ill*)) OR (suicide NEAR/1 assist*)))  abstract("End of life" OR "Supportive care") OR title("End of life" OR "Supportive care") OR subject("End of life" OR "Supportive care") |
| **Concept 1 : mixed strategy (S3)** | **S1 OR S2** |
| **Concept 2 : thesaurus strategy (S4)** | Mainsubject.Exact("girls education" OR "adolescents" OR "boys" OR "children & youth" OR "girls" OR "boys education" OR "children" OR "teenagers") |
| **Concept 2 : keywords strategy (S5)** | abstract(Youth* OR Child* OR Boy* OR Girl* OR Kid OR Kids OR Adolescen* OR Teen*) OR title(Youth* OR Child* OR Boy* OR Girl* OR Kid OR Kids OR Adolescen* OR Teen*) OR subject(Youth* OR Child* OR Boy* OR Girl* OR Kid OR Kids OR Adolescen* OR Teen*) |
| **Concept 2 : mixed strategy (S6)** | **S4 OR S5** |
| **Concept 3 : thesaurus strategy (S7)** | Mainsubject.Exact("elementary school teachers" OR "curriculum development" OR "elementary school students" OR "kindergarten" OR "secondary schools" OR "elementary education" OR "kindergarten teachers" OR "secondary education" OR "secondary school teachers" OR "secondary school students" OR "elementary schools" OR "kindergarten students") |
| **Concept 3 : keywords strategy (S8)** | abstract(School* or Kindergarten* or Curriculum* or Teacher* or Pupil*) OR title(School* or Kindergarten* or Curriculum* or Teacher* or Pupil*) OR subject(School* or Kindergarten* or Curriculum* or Teacher* or Pupil*)  abstract((Education OR Student*) NEAR/1 (Primary OR Secondary OR Elementary))) OR title((Education OR Student*) NEAR/1 (Primary OR Secondary OR Elementary))) OR subject((Education OR Student*) NEAR/1 (Primary OR Secondary OR Elementary))) |
| **Concept 3 : mixed strategy (S9)** | **S7 OR S8** |
| **Final strategy** | **(S3 AND S6 AND S9)** |
